# Towards Automated Analysis of Grain Spikes in Greenhouse Images Using Neural Network Approaches: A Comparative Investigation of Six Methods

**DOI:** 10.3390/s21227441

**Published:** 2021-11-09

**Authors:** Sajid Ullah, Michael Henke, Narendra Narisetti, Klára Panzarová, Martin Trtílek, Jan Hejatko, Evgeny Gladilin

**Affiliations:** 1 Plant Sciences Core Facility, CEITEC—Central European Institute of Technology, Masaryk University, 60200 Brno, Czech Republic; mhenke@uni-goettingen.de (M.H.); hejatko@sci.muni.cz (J.H.); 2 Leibniz Institute of Plant Genetics and Crop Plant Research (IPK), 06466 Gatersleben, Germany; narisetti@ipk-gatersleben.de; 3 PSI (Photon Systems Instruments), spol. s r.o., 66424 Drasov, Czech Republic; panzarova@psi.cz (K.P.); martin@psi.cz (M.T.)

**Keywords:** high-throughput plant image analysis, spike detection, spike segmentation, deep learning, automated plant phenotyping

## Abstract

Automated analysis of small and optically variable plant organs, such as grain spikes, is highly demanded in quantitative plant science and breeding. Previous works primarily focused on the detection of prominently visible spikes emerging on the top of the grain plants growing in field conditions. However, accurate and automated analysis of all fully and partially visible spikes in greenhouse images renders a more challenging task, which was rarely addressed in the past. A particular difficulty for image analysis is represented by leaf-covered, occluded but also matured spikes of bushy crop cultivars that can hardly be differentiated from the remaining plant biomass. To address the challenge of automated analysis of arbitrary spike phenotypes in different grain crops and optical setups, here, we performed a comparative investigation of six neural network methods for pattern detection and segmentation in RGB images, including five deep and one shallow neural network. Our experimental results demonstrate that advanced deep learning methods show superior performance, achieving over 90% accuracy by detection and segmentation of spikes in wheat, barley and rye images. However, spike detection in new crop phenotypes can be performed more accurately than segmentation. Furthermore, the detection and segmentation of matured, partially visible and occluded spikes, for which phenotypes substantially deviate from the training set of regular spikes, still represent a challenge to neural network models trained on a limited set of a few hundreds of manually labeled ground truth images. Limitations and further potential improvements of the presented algorithmic frameworks for spike image analysis are discussed. Besides theoretical and experimental investigations, we provide a GUI-based tool (SpikeApp), which shows the application of pre-trained neural networks to fully automate spike detection, segmentation and phenotyping in images of greenhouse-grown plants.

## 1. Introduction

The derivation of reliable quantitative traits (QTs), such as morphological and developmental features, became the method of choice by investigation of the effects of biotic and abiotic factors on plant growth and grain yield [1]. However, the high variability of optical setups and plant appearance turned out to render a non-trivial task for image-based phenotyping, which represents one of the major bottlenecks of quantitative plant science [2,3]. In addition to assessment of the overall plant biomass and structure, the detection and quantification of plant organs, such as wheat ears and spikes, is of particular interest for biologists and breeders.

**Figure 1:**
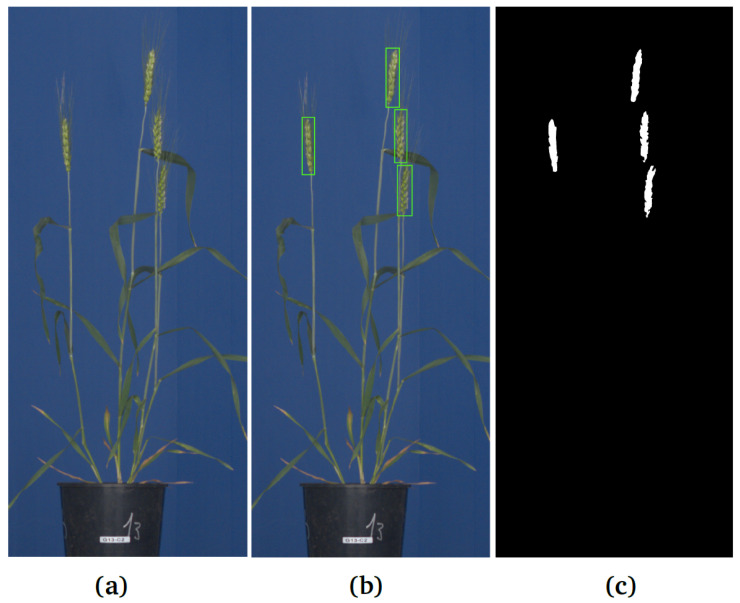
Example of spike detection and segmentation in greenhouse wheat images: (**a**) RGB visible light image of a matured wheat plant, (**b**) detection of spikes by rectangular bounding boxes, (**c**) pixel-wise segmentation of spikes.

The predominant majority of previous works were focused on the analysis of spikes visible on the top of plants grown under field conditions, where researchers were primarily interested in assessing spike counts and density per square area [3,4,5]. In contrast to field images, where spikes are only visible on the top of grain crops, greenhouse images of single plants acquired from different rotational angles in side view potentially enable to assess the amount and phenotype of all spikes, including spikes that emerge not only on the top, but also within the mass of plant leaves, as is often the case for many European wheat cultivars. In general, the high-throughput phenotyping of plants in a controlled greenhouse environment is used for the investigation of effects of environmental conditions, such as drought stress, temperature, light intensity as well as their fluctuations [6,7]. Furthermore, detection of spikes in images of greenhouse-grown plants is of interest for subsequent remote screening of grain yield and development, using X-ray imaging, which requires the precise location of spikes in the image. However, even in a controlled greenhouse environment, spikes can be partially covered by leaves and/or occluded together, which hampers their straightforward detection and phenotyping. Depending on the particular research goals, biologists are, in general, interested in automation of two major tasks: (i) detection/localization/counting and (ii) pixel-wise segmentation of spikes. See examples in Figure 1.

The latter enables the assessment of such important traits as spike area (biomass), shape, color, and texture, which is otherwise not accessible by means of pattern detection methods.

A plethora of conventional and modern methods for spike image analysis in different optical and environmental setups for different biological tasks was developed in the past. From the summary of existing approaches to spike image analysis in Table 1, it is evident that the majority of previous works focused on spike image analysis under field conditions. Furthermore, different measures were used in different studies, including average precision (AP), accuracy of the confusion matrix (A) and F1-score (also known as the Dice coefficient).

Grillo et al. applied image analysis techniques to identify wheat landraces based on glume phenotypes by statistical analysis of morpho-colorimetric descriptors [8]. Alharbi et al. [5] detected wheat ears by transforming raw plant images, using the color index of vegetation extraction (CIVE) and performed the clustering of pixel features extracted in the CIVE domain, using k-means. CIVE uses principal component analysis for the estimation of biomass. Tan et al. applied support vector machine (SVM) and k-nearest neighborhood for wheat spike recognition on pre-segmented spike regions and super-pixels that were generated by simple linear iterative clustering. Bi et al. designed different architectures of a 3-layer neural network based on the number of hidden layer nodes to classify four wheat varieties of a sole-spike image to extract the spike traits, such as the awn number, the average awn length, and the spike length [9]. Misra et al. presented SpikeSegNet, which performs spike detection with two cascaded feature networks: local patch extraction and a global mask refinement network [10]. Hasan et al. achieved spike detection and counting with R-CNN obtaining a F1 score of 0.95 on 20 wheat field images, having an average of 70–80 spikes per image [3]. Pound et al. implemented a deep neural network (DNN) for the identification of spikelets and their counting [11]. Most of the above methods were developed for field image analysis and restricted to a particular subset of image data and imaging modalities. The source code of the algorithmic implementations or deployed tools are rarely provided for reproducing the results and routine application.

Only a few works are known to deal with detailed analysis of spikes images acquired from greenhouse plant phenotyping experiments. Qiongyan et al. presented a spike segmentation framework based on artificial (shallow) neural networks (ANNs), which was trained and evaluated only on images of wheat species exhibiting spikes on the top of the plant (further termed ‘top spikes’) and almost no leaf-covered (‘inner’) or occluded spikes [12]. In our previous work, we extended the ANN approach to the detection of more difficult bushy European wheat phenotypes [13]. Improvements introduced to the shallow ANN architecture, such as Frangi line filters, could enhance the final segmentation results; however, this ANN framework still requires substantial efforts, such as parameter adjustment by application to new image data. In particular, the improved ANN performed well on detecting spikes in crops with relatively low biomass and low yielding but showed limitations when applied to high biomass and high yielding wheat images. In these phenotypes, spikes emerge within the mass of leaves. In such cases, the Frangi filters did not suffice for filtering out spike regions from wrongly segmented leave and tiller edges. Further, this method also employed a morphological reconstruction step to compensate for the reduced area prediction as a result of pure performance on the spike boundaries. This step can be mitigated if the neural network architecture is deployed to reconstruct or upsample the feature samples, such as in the case of a encoder–decoder architecture.

The majority of previous works is typically evaluated on a particular, typically limited, set of images such that the generalizability of one or other method by application to a new experimental setup is difficult to assess. In fact, spikes may exhibit different shape, colors and textures depending on the plant species, developmental stage and experimental environment, which makes the generalization of spike detection/segmentation, especially using conventional methods, a challenging problem. In the absence of unique and robust features for the classification of fore- and background regions, already the very first task of appropriate feature definition is not trivial and has to be approached in a very general manner. Deep learning methods allow to address this task by performing the automated search and selection of features. With the success of AlexNet [14], significantly more robust and accurate results of image segmentation were achieved in a widely automated manner as compared to traditional classification techniques based on a pre-defined set of features. The top performance of DNNs on a benchmark data set, i.e., VOC 2007-12 (visual object classes) and MS COCO (common objects in context), is attributed to the automated feature extraction of classifier and pixel-wise segmentation [15,16]. Meanwhile, numerous DNN architectures were reported for the frequently demanded tasks of pattern detection and image segmentation. However, studies demonstrating the performance of different DNNs in application to plants, plant organs and, in particular, spike detection/segmentation are quite rare. In view of the generally known challenges by analysis of small and optically variable structures, here, we decided to approach the problem of detection/segmentation of diverse (‘top’, ‘leaf-covered’, and ‘occluded’) spikes of different cereal plants (wheat, barley, and rye) by investigating and comparing the performance of six different machine learning frameworks, including three detection deep neural networks (DNNs), such as single shot multibox detector (SSD), faster-RCNN, and YOLOv3/v4, as well as two segmentation DNNs (U-Net, DeepLabv3+) and one conventional shallow ANN.

The optical appearance of spikes changes through the life cycle of cereal plants from vegetative to reproductive stage. The detection of emergent spikes provides important quantitative traits of plant development and yield to plant breeders and biologists. The localization of spikes in the mass of leaves is particularly difficult in the early reproductive stage as well as during the harvesting period when both spikes and leaves exhibit similar color fingerprints. The multi-view imaging systems in greenhouse photo chambers provide not only side, but also top view images, where spikes often exhibit a similar profile as in the side view images but with arbitrary spatial orientation. Consequently, we were interested in evaluating whether spike detection models trained on the majority of side view images can also be applied to the detection of differently oriented spike patterns in top view images. Furthermore, this study investigates the generalizability of detection/segmentation models trained on a particular crop cultivar (e.g., wheat) by application to other crop species (e.g., barley and rye).

Our work provides comprehensive insights into the entire process of data preparation, model training, and evaluation, and addresses the central question what can be achieved with the above state-of-the-art NN methods, using a typically limited amount of manually annotated ground truth images in terms of accuracy and generalizability. In addition to the experimental investigations, we provide potential end users with a GUI-based tool (SpikeApp) that demonstrates the automated detection, segmentation and phenotyping of spikes in greenhouse-grown plants, using three pre-trained neural network models, including U-Net, YOLOv3 and shallow ANN.

## 2. Methods

### 2.1. Image Acquisition

Wheat plant images were acquired from the high-throughput greenhouse phenotyping system PlantScreen^TM^ of Photon Systems Instruments (PSI) (https://psi.cz/, accessed on 1 January 2020). Twenty-two cultivars of Central European wheat were imaged in vegetative and reproductive stages, taken in the PSI photo chamber. Out of the 22 crop cultivars, 19 were selected for spike detection and the segmentation task. An overview of the wheat cultivars analyzed in this work, including the number of RGB visible light images of each cultivar, is provided in Table 2.

The plant images for the experiment were captured in the side view from two rotational angles (0°and 90°). The images were taken in the same resolution, 2560 × 2976, using the uniform blue background. The entire set of original images with the test set used in this study are available at https://ag-ba.ipk-gatersleben.de/ghimgs.zip (accessed on 1 November 2021).

### 2.2. Data Set Preparation

The data set of 292 images was divided into training and test sets in the proportion 80:20, regardless of the spike numbers, spatial position and orientation. All images were manually annotated for the training and testing of the spike detection and segmentation models.

Consequently, 234 images with the total number of 600 spikes were used for training the neural network models. The training set was extended with the inclusion of YSYC wheat images as shown in Table 3. The deep convolution neural network (DNN) used for spike detection was trained on original images of the size 2560 × 2976 stored in the PNG format. The training data set was reiterated for reduced resolution of 800 × 600. The multi-resolution testing of DNNs was necessary to ensure that the DNN can preserve high-frequency information of the spike boundary at a lower resolution. The annotations for the spike detection were done with LabelImg [17] by drawing a bounding box around each spike and subsequently saved as a ∗.xml file as required for Faster-RCNN and SSD. For YOLO, the annotations were converted to a ∗.json file. The spike labeling for segmentation was accomplished by GIMP image processing software with Free Select tool and Bucket Fill. The labeled structures were saved as grayscale images. The segmentation was regarded as binary pixel-wise labeling, with the spike region having the value of 1, and the non-spike region having the value of 0.

The training set comprises 234 wheat images from 19 cultivars. A total of 219 wheat plants were imaged through their life cycles until the spikes were mature for harvesting. Out of the 234 images, 33% of the plant images were taken from two direction side views (0°and 90°). The testing set comprises 58 images, including 8 images that contain spikes occluded by the leaves or, in some cases, the stem of the plant.

**Figure 2:**
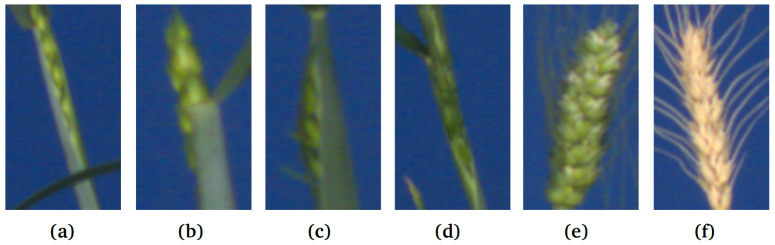
Examples of spike ROIs from the test image set: (**a**–**d**) emergent, partially visible spikes vs. (**e**,**f**) matured spikes (GSGC, YSYC).

The training set contains 203 images of Green Spike and Green Canopy (GSGC), 27 images of Yellow Spike and Yellow Canopy (YSYC) and 4 negative training (no spikes) images. These training images have fewer leaves in the plant, compared to the high-yielding Central European wheat plants (in the generalization test) in which the challenge is to detect spikes that exhibit colors similar to the leaves. The total count of spikes in the test set is 125. The test images contain not just the mature spike in the reproductive cycle of wheat, but also include the example of emergent and partially visible spikes; see Figure 2. These spikes were distributed over 12 images in the testing set and the total count was 18. The goal was to see whether the trained model can detect the high-frequency boundary in those spikes. The output of the spike detection represents a list of bounding boxes with class (spike) probability. The spike segmentation output is labeled as a spike or background (non-spike) region. In the test set, the numbers of GSGC, YSYC and non-spike images are 49, 7 and 2, respectively.

### 2.3. Spike Detection DNN Models

In this section, we describe DNN models for spike detection starting from deep to deeper neural network: SSD, faster-RCNN and YOLOv3/v4.

#### 2.3.1. Single Shot Multibox Detector

SSD is a feed-forward convolutional network that makes multiple predictions for the bounding box of spikes crossover different scales [18]. SSD has the characteristics of generating region proposal like YOLOv3. The single stage detector divides the image into grid cells, and each cell has a likelihood of a spike being located in it. In the case of multiple objects in a grid, the SSD in-training process deploys a pre-defined aspect ratio of anchor boxes and produces a score for each object in each box.

#### 2.3.2. Faster-RCNN

Selective search was used successfully for region proposals generation [19]. On the other hand, faster RCNN deploys a small DNN for feature extraction for a region proposal network (RPN). In recent years, RPN was used prior to the main object detector, which produces candidate objects with an objectness score for object detection, and was implemented successfully in many publicly available data sets [20]. In this work, we have implemented a two-stage cascaded detection Faster-RCNN framework [21]. In the first stage, multiple objects are proposed prior to extracting features for the main detector. These proposals are spike or background. Its deployment improves the classifier accuracy, but this improvement come at the expense of an increase in computational resources. The RPN is implemented as a fully convolutional network and works as a mini-network trained end-to-end with a main object detector. RPN and region-based object detection DNN share the same convolution feature. The computational speed of RPN compared to the selective search space is 10 ms to 0.2 s per image [21]. The features in the convolution layers are shared in RPNs and DNN object detectors. The RPNs are fed directly to the DNN object detector. The DNN initializes the weights in RPNs with zero-mean Gaussian distribution and standard deviation 0.01. In the first stage of processing, 100 spike proposals were extracted for features and regression. Each anchor/proposal generated has either a positive anchor label or negative anchor label. The positive label is assigned to those which have the highest IoU with ground truth or an IoU overlap higher than 0.7, whereas the negative anchor label is one in which the IoU is lower than 0.3. The RPN computes the features map with six different anchor sizes, differentiated by the aspect ratio, which is initialized at the beginning of the training process. Therefore, each regressor is responsible for extracting weight in a separate spatial size (n×n) on the feature map. The loss (softmax loss for classification and L1 loss for regression) in RPN is calculated in a mini-batch to mitigate the bias towards background space, as it is dominated by negative anchors. The negative anchors are background samples, compared to the foreground, which occupies a smaller spatial size. This is done to ensure that the classifier is not biased towards the over-sampled anchors from the background.

**Figure 3:**
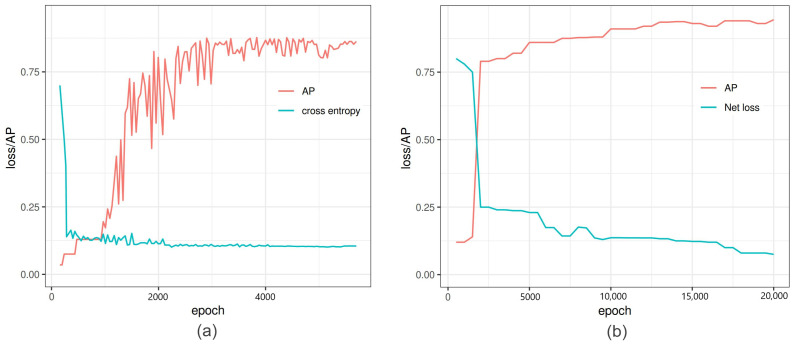
Comparison of performance of Faster-RCNN vs. YOLOv3: (**a**) Faster-RCNN in-training loss and average precision (AP) versus iterations of Faster-RCNN. At 6000 iterations, the binary cross entropy loss is minimized with high AP, and further training increases the loss and AP altogether. (**b**) YOLOv3 in-training binary cross entropy loss and average precision versus the epoch number.

In the second stage of Faster-RCNN, exponential decay is used as a learning parameter for training images. The features computed in RPN are passed through an ROI pooling layer and turned into a feature vector for the fully connected layer in the main detector. Finally, a softmax layer produces a binary output assigned with a set of class probabilities, and the regressor computes a bounding box with accurate coordinates. Typical curves of in-training loss and average precision during the training process of Faster-RCNN are depicted in Figure 3a.

#### 2.3.3. YOLOv3 and YOLOv4

The YOLOv3 and its v4 variant differ from the region of proposed networks by the selection of an initial proposal for the feature map extraction. YOLOv3 divides the in-training image into a fixed number of S×S grid. The class label is predicted for a single object in the grid cell [22]. For each grid cell, the fully connected layers output the bounding box and confidence score computed in a single forward pass from conditional class probabilities. The objectness score for the bounding box is computed using logistic regression. The YOLOv3 variant is Faster for real-time object detectors by dividing the image into a fixed grid. As the backbone in YOLOv3, we implemented Darknet53, while for YOLOv4, we took CSPDarknet53. In YOLOv4, the Mish activation function is used on the output convolution layer in the feature extractor and detector [23]. The training loss for class prediction used is binary cross-entropy, while sum squared error is used for the calculating the loss of bounding box prediction. The network has cascaded 3×3 and 1×1 convolutional layers. The skip connection, which bypasses certain layers, results in uninterrupted gradient flow. The size of the layer skipping is greater in Darknet53 than its predecessor Darknet19. The shortcut connection skips the detection layer that does not decrease the loss on those layers. The spike prediction is done across three scales in detection layers. The bounding boxes are predicted with a dimension cluster. The output 4D tensor prediction of the bounding box consists of four coordinates: $t_{x}$, $t_{y}$, $t_{w}$ and $t_{h}$. Logistic regression is used to compute the objectness score for every bounding box. If the overlap between the predicted bounding box and ground truth is 0.5, the class probability of the bounding box has a confidence of 1. Logistic classifier is deployed at the prediction layer for classification.

The efficient use of defining objects in individual cell gives it a competitive edge over other state-of-the-art DNNs, for example, ResNet101 and ResNet152, particularly for real-time application [24]. The training process of YOLOv3 is depicted in Figure 3b. The network was trained on an image size of 2560 × 2976. The training process took nine hours.

One of the improvements of YOLOv4 over YOLOv3 is the introduction of mosaic image enhancement. The image augmentation of CutOut, MixUp and CutMix were implemented. The loss function used in training of the YOLOv4 includes classification loss ($L_{class}$), confidence loss ($L_{confidence}$) and bounding box position loss ($L_{cIoU}$) [23].

(1) $$Net\;loss\;=\;L_{class}+L_{confidence}+L_{cIoU}\;.$$

### 2.4. Spike Segmentation Models

The section gives a description of spike segmentation NNs, including two DNNs (U-Net, DeepLabv3+) and a shallow ANN.

#### 2.4.1. Shallow Artificial Neural Network

The shallow artificial neural network (ANN) approach from [12] with extensions introduced in [13] was retrained with ground truth segmentation data for leaf and spike patterns from the training set. The texture law energy, well known from several previous works [9,25,26], was used in this approach as the main feature. As a pre-processing step, the grayscale image is converted to wavelet discrete wavelet transform (DWT) using the Haar basis function. The DWT is used as input to shallow ANN. In the first feature extraction step, nine 3×3 convolution masks of size 2n+1 are convolved with the original image *I*. The convolutional equation for this step is given as follows:

(2) $$F\left(i,j\right)\;=\;\left(A*I\right)\left(i,j\right)\sum_{p=a}^{a}\sum_{p=-a}^{a}I\left(i+p,j+l\right)\;,$$

where *I* represents the image, and *A* represents one of three masks. Details are described in [13]. In the second step, the mean deviation around a pixel is computed by macro-windowing operation of size (2n+1)(2n+1) on the neighborhood of every pixel. It is computed as follows:

(3) $$E\left(i,j\right)\;=\;\frac{1}{\left(2n+1\right)}\sum_{p=i-n}^{i+n}\sum_{l=j-n}^{j+n}\left|F\left(p,l\right)\right|\;,$$

where *E* symbolizes the energy texture measure. Finally, the boundaries obtained from ANN are filtered using a multiscale Frangi filter to eliminate noisy edges as described in [13].

#### 2.4.2. U-Net

In this work, the U-Net architecture from [27] was adapted to process RGB spike images. U-Net consists of a down sampling path in which the feature map is doubled in the encoder block, while image size is reduced by half. Each of the five blocks of the contracting path consists of a consecutive 3×3conv layer and followed by a Maxpool layer. The plateau block has also a pair of consecutive conv layers without a Maxpool layer. The layers in the expansive path are concatenated with the corresponding layer for the feature map in the contracting path, which makes the prediction boundary of the object more accurate. In the expansive path, the size of the image is restored in each transposedconv block. The feature map from conv layer is succeeded by RELU and the batch normalized layer. The final layer is 1×1conv, a layer with 1 filter which produces the output binary pixels. The U-Net is a fully convolutional network without any dense layers. In order to enable training the U-Net model on the original image resolution, including important high-frequency information, the original images were cropped into masks of 256 × 256 size. Using the full-size original images was not possible, due to the limitations of our GPU resources. Since spikes occupy only very small image regions, the usage of masks helped to overcome limitations by processing the full-size images while preserving the high-frequency information. To mitigate the class imbalance issue and to remove the frames that solely have a blue background, we maintained the ratio of spike vs. non spike (frame) regions as 1:1.

#### 2.4.3. DeepLabv3+

DeepLabv3+ is a state-of-the-art segmentation model that has shown a relatively high mIoU of 0.89 on PASCAL VOC 2012 [28]. The performance improvement is particularly attributed to the Atrous Spatial Pyramid Pooling (ASPP) module, which obtains contextual information on multi-scale at several atrous convolution rates. In DeepLabv3+, atrous convolution is an integrated part of the network backbone. Holschneider et al. [29] employed atrous convolution to mitigate the reduction in spatial resolution of feature responses. The input images are processed using the network backbone. The output is elicited from each location *i* and filter weight *w*. The atrous convolution is processed over the feature map. The notation for atrous convolution signal is similar to that used in [30] for location *i* and filter weight *w*. When atrous convolution is applied over feature map x, the output y is defined as follows:

(4) $$y\left[i\right]\;=\;\sum_{k=1}^{K}\left[i+r.k\right]w\left[k\right]\;,$$

where *r* denotes the rate at which the input signal is sampled. The feature response is controlled by atrous convolution. The output stride is defined as the ratio of the input spatial resolution to the output spatial resolution of the feature map. A large-range link is established between the network backbone and multiple-scale feature extraction modules: ASPP and dense prediction cell (DPC). The depth-wise separable convolution operates convolution on each channel followed by point-wise convolution (1×1), which superimposes the feature signal from the individual channel.

In the decoder part, the features are bilinearly upsampled, the output of which is convolved with 1×1 convolution and then concatenated with low-level features. Another 3×3 convolution is operated on the feature map followed by bilinear upsampling, and the output is binary semantic labels. Here, we modified and implemented publicly available DeepLabv3+ code for training and evaluation on our spike image data set [28].

By training U-Net and DeepLabv3+, conventional augmentation techniques, including rotation [−30 30], horizontal flip, and image brightness change [0.5 1.5] were adopted. The ratio of the augmented images has the same proportion of GSGC:YSYC and also the non-spike images as previously used in our training set for the detection of DNNs.

### 2.5. Evaluation of Spike Detection Models

The DNNs deployed in this work are evaluated by mAP, which is computed as a weighted mean of precision at different threshold values of recalls. The average precision is computed as the mean precision value at 11 equally spaced recall levels (0, 0.1, 0.2, …, 1). On the PASCAL VOC2007 evaluation measure, mAP is 0.5 when the IoU between the prediction bounding box and ground truth box is 0.5. As a result, mAP has a global view of the precision–recall curve. For every recall, the maximum precision is taken. In COCO, mAP is the 101-interpolated point computed over 10 different IoU (0.5:0.05:0.95) with a step size of 0.05. The final value of mAP is averaged over the classes. In this work, we evaluate the three detection DNNs (SSD, YOLOv3, and Faster-RCNN) and three segmentation models (ANN, U-Net, and DeepLabv3+) on a test set of 58 images. Precision (*P*), recall (*R*), accuracy (*A*) and F1 measures are calculated based on standard detection benchmarks, such as PASCAL VOC and COCO. A positive prediction value/precision is the number of true spike frames correctly classified as a spike:

(5) $$P\;=\;\frac{TP}{TP+FP}\;.$$

The true positive rate/recall is the number of spikes in the test image that was localized with the bounding box (IoU ≥ 0.5):

(6) $$R\;=\;\frac{TP}{TP+FN}\;,$$

(7) $$A\;=\;\frac{TP+TN}{TP+TN+FP+FN}\;.$$

The model robustness is quantified by calculating the harmonic mean of precision and recall as follows:

(8) $$F1\;=\;2\frac{P*R}{P+R}\;.$$

We have evaluated our data set with commonly used metrics for object detection, such as PASCAL VOC and COCO detection measures. The mAP used to evaluate the localization and class confidence of spike is calculated as follows:

(9) $$mAP\;=\;\frac{1}{N}\sum_{i=1}^{N}AP_{i}\;.$$

In PASCAL VOC 2007, the average precision, AP is calculated at a single IoU value. In the COCO evaluation, which has a more stringent evaluation measure than PASCAL VOC, AP is calculated at 10 different IoU thresholds (0.5:0.05:0.95), while the final mAP of DNN is averaged over the 10 IoU threshold values. The mean of the average precision is computed on both classes: spike and background. The binary output from the segmentation task is evaluated by the Dice coefficient score. A binary mask of prediction is the output with zeros for non-spike pixels and ones for spike pixels. The F1 score for segmentation, in contrast to the spike detection, is done at the pixel level. We also evaluated the test set with the IoU/Jaccard index. Both evaluation measures for segmentation are positively correlated.

### 2.6. Evaluation of Spike Segmentation Models

The performance of the segmentation method was quantified by a commonly used evaluation measure of the boundary F1 score and intersection-over-union (IoU) also known as the Jaccard index. The average Dice coefficient, aDC, is another metric used for pixel labeling, calculated by Equation (8) and then taking the average of both regions. Given the set of class of ground truth spike and background label and predicted binary labels, the IoU metric is defined as the number of pixels common between the ground truth and predicted mask divided by the total number of pixels present across both masks. The mean IoU represents the average intersection over the union of spike and non-spike region. The evaluation of predicted pixel of object is compared with ground truth computed by Equation (10). The output of the segmentation network is binary pixels (spike pixel = 1; non-spike pixel = 0).

(10) $$IoU\;=\;\frac{TP}{TP+FP+FN}\;.$$

Spike detection and segmentation experiments were run on a Linux operating system with Ryzen 7 3800X using 80 GB RAM and RTX 2080Ti (11GB VRAM.)

## 3. Results

The results of this study are structured and presented as follows.

- First, the performance of neural network models for detection and segmentation of wheat spikes in side view greenhouse images was investigated.
- Then, the NN models trained on side view images of wheat plants were applied to other crops (barley and rye).
- Finally, spike detection and segmentation models trained on side view images of wheat plants were validated by application to side and top view images of other, more bushy wheat cultivars acquired from another greenhouse facility.

The above tests aimed to evaluate the performance of different spike detection and segmentation models trained on a particular set of images (namely, side view wheat plants) by application to (i) images of the same and (ii) different crop cultivars screened in the same facility as well as to (iii) images of phenotypically more distant wheat cultivars from another greenhouse facility.

### 3.1. Spike Detection Experiments

The detection of spike patterns was performed, using SSD, Faster-RCNN and YOLOv3/v4 DNN models trained on a data set of totally 234 images as described above. Table 4 summarizes the evaluation of all spike detection DNN models on PASCAL VOC ($AP_{0.5}$) and COCO detection metrics ($AP_{0.5:0.95}$).

#### 3.1.1. Spike Detection Using SSD

The SSD model was trained using stochastic gradient descent (SGD) with an initial learning rate of 0.001, momentum of 0.9, weight decay of 0.0005, and batch size of 32. The SSD was trained for 22,000 iterations, which took 10 hours on the GPU. On that iteration, the loss was minimized on validation data and further training overfit the model. Out of three DNNs, SSD performed with the lowest average precision. In this regard, our observation confirmed the previous findings from [31] that SSD does not perform well on small objects, such as spikes in our case.

#### 3.1.2. Spike Detection Using Faster-RCNN

Faster-RCNN was trained for 6000 iterations with binary cross-entropy and learning rate scheduling strategy of exponential decay. The network was developed with an Adam optimizer with a momentum of 0.9. In training, a batch size was set to 6. Inception v2 was taken as the backbone for the main detector. Faster-RCNN training was trained for 6500 iterations for 9 hours on GPU. Around 6000, the loss and in-training AP was lowered enough so that the training process stopped. Then, the training was performed on the training set of images with 800 × 600 resolution. The number of false positives was 6 out of the total number of spikes in the test set (125). The number of true positive spikes was 100 on lower resolution, while on the original image resolution, the number of detected spikes was 119 with a false positive of 3. Remarkably, the set of false positive spikes comprised mostly GSGC test images. The inference time of the Faster-RCNN was 0.25 frame/s on the original image resolution. The test images comprised side viewing spikes only. There was no difference in performance by using the dropping learning rate on the fixed iteration.

#### 3.1.3. Spike Detection Using YOLOv3/v4

The third DNN we trained was YOLOv3, which performed well on the VOC 2007, 2012, and MS COCO data sets. YOLOv3 was trained using stochastic gradient descent (SGD) algorithm for nine hours with a batch size of 64 and subdivision of 16. The input height and width to the network was kept on resolution of 416 × 416. The learning parameter was 0.001 with a decay factor of 0.0005 and momentum of 0.9. The inference on the test image was done with non-maximal suppression to exclude multiple prediction on spikes. In addition, YOLOv4 was trained for 20,000 epochs with polynomial decay learning rate scheduling strategy starting at 0.1 with a decay of 0.005, momentum of 0.9 and mini-batch size of 8. All detection models achieved comparatively better result ($AP_{0.5}>0.75$) with Faster-RCNN outperforming SSD and YOLOv3/v4 models by 20% and 1.06%, respectively. The mAP of YOLOv3 and YOLOv4 were similar on $AP_{0.5}$, but YOLOv4 showed better precision on $AP_{0.50:0.95}$. On low resolution images (800 × 600), the $AP_{0.5}$ of the DNNs decreased by 3–5%, which indicates that the DNNs did not extract features for the high-frequency spike region in lower resolution images. The inference time of the YOLOv3 on test image was 2.30 frame/s, which is close to the average value of YOLOv4. Faster-RCNN consumed the most memory (45 GB) for training, followed by SSD (20 GB) and YOLOv3/v4 (8 GB).

**Figure 4:**
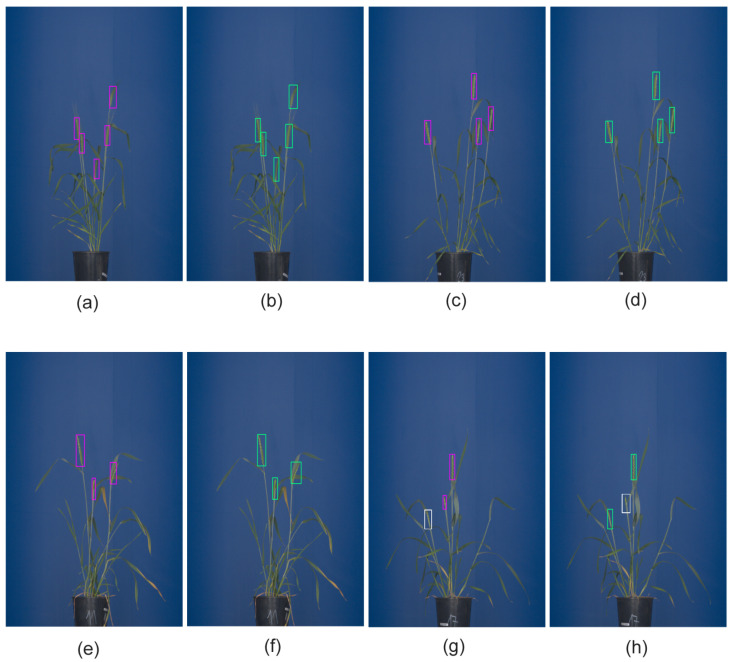
The detection of grain spikes using pre-trained Faster-RCNN (green bounding boxes) and YOLO (pink bounding boxes) DNN: (**a**–**d**) examples of detection of top wheat spikes, (**e**–**h**) examples of detection of emergent wheat spikes White bounding boxes indicate spikes that were not detected by the DNN classifier in this particular image.

Among the above three detecting DNNs, the Faster-RCNN and YOLOv3 models showed significantly better performance with mAP over 0.94, compared to SSD, having a modest mAP of 0.78. The best models were selected based on $AP_{0.5}$. Figure 4a–d shows examples of Faster-RCNN and YOLOv3 performance on test images of matured spikes. Such spikes were localized by Faster-RCNN, YOLOv3 and YOLOv4 with $AP_{0.5}=0.99$. However, not all spikes show the same prominent optical appearance as matured spikes growing on the top of the plant. In addition to such clearly visible ‘top spikes’, some matured spikes may appear in the middle of the mass of leaves that have a similar color fingerprint. Yet another category of spikes represents emergent and occluded spikes that differ from matured spikes with regard to both the effectively visible area and texture. The different optical appearance of such spikes led to the decreased performance of DNNs, with YOLOv4 achieving the higher $AP_{0.5}=0.805$ followed by Faster-RCNN $AP_{0.5}=0.800$. Examples of the detection of occluded and emergent spikes using Faster-RCNN and YOLO are shown in Figure 4e–h. In YOLOv4, augmentation includes such transformations as Random Erase, CutMix and MixUp. Augmentation helps to avoid overfitting and works as a regularization for the model. The number of augmented images used in pattern detection DNNs is summarized in Table 4.

**Figure 5:**
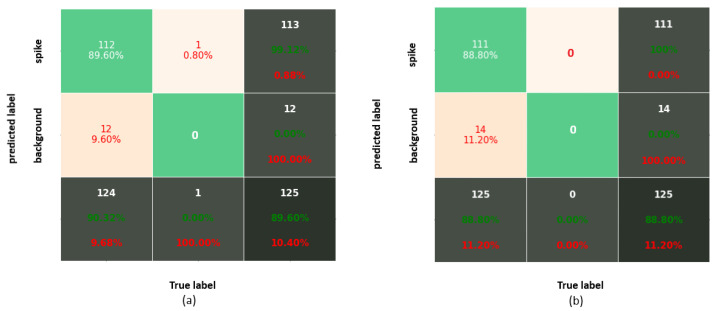
Confusion matrix corresponding to IoU = 0.75 for (**a**) Faster-RCNN and (**b**) YOLOv3.

Performance measures of all DNNs, including AP, accuracy and average probability for matured spikes appearing on the top of the plant, in the middle of the mass of leaves (‘inner spikes’) as well as partially visible occluded/emergent spikes are summarized in Table 5. Figure 5 shows the cumulative confusion matrix for Faster-RCNN and YOLOv3 detection models.

### 3.2. Spike Segmentation Experiments

**Figure 6:**
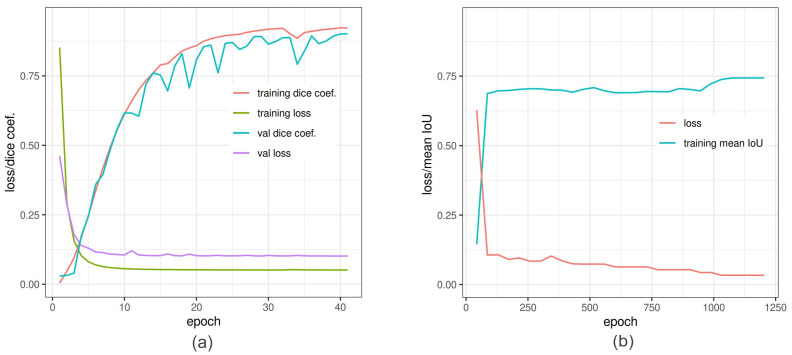
In-training accuracy of U-Net and DeepLabv3+ versus epochs: (**a**) Dice coefficient (red line) and binary cross-entropy (green line) reached pleateau around 35 epochs. The training was also validated by Dice coefficient (light sea-green line) and loss (purple line) to avoid overfitting. (**b**) Training of DeepLabv3+ is depicted as function of mean IoU and net loss. The loss converge around 1200 epochs.

The segmentation of spike images was performed, using a shallow ANN and two DNN models (U-Net and DeepLabv3+). The evaluation measures of U-Net and Deeplabv3+ in the training process are shown in Figure 6. Table 6 summarizes the performance of all three spike segmentation models on the test set of spike images.

#### 3.2.1. Spike Segmentation Using ANN

The training of ANN was performed on manually segmented ground truth images where spike pixels were assigned the label value of 1 and the remaining background pixels, the value of 0. In the test set of spike images, the shallow ANN showed a satisfactory performance with aDC of 0.76 and Jaccard index of 0.61.

#### 3.2.2. Spike Segmentation Using U-Net

The training process was performed with RELU as an activation function. In the output of the U-Net prediction, the value of 0 is assigned to background pixels and the value of 1 to spike pixels, resulting in binary pixel-wise image segmentation. The U-Net model was optimized by the Adam optimizer [32] with the variable learning rate scheduling decreasing with each epoch from $3\times 10^{-3}$ to $1\times 10^{-5}$.

U-Net was trained on RTX 2080Ti for 45 epochs on 256 × 256 frames with a batch size of 32. In the training process, it was validated by binary cross entropy loss and Dice coefficient on 45 images (0.1 *training set) of the validation set. No improvement was observed when Tversky loss was used for the training process [33]. On the test set of spike images, the U-Net reached aDC of 0.9 and Jaccard index of 0.84.

#### 3.2.3. Spike Segmentation Using DeepLabv3+

**Figure 7:**
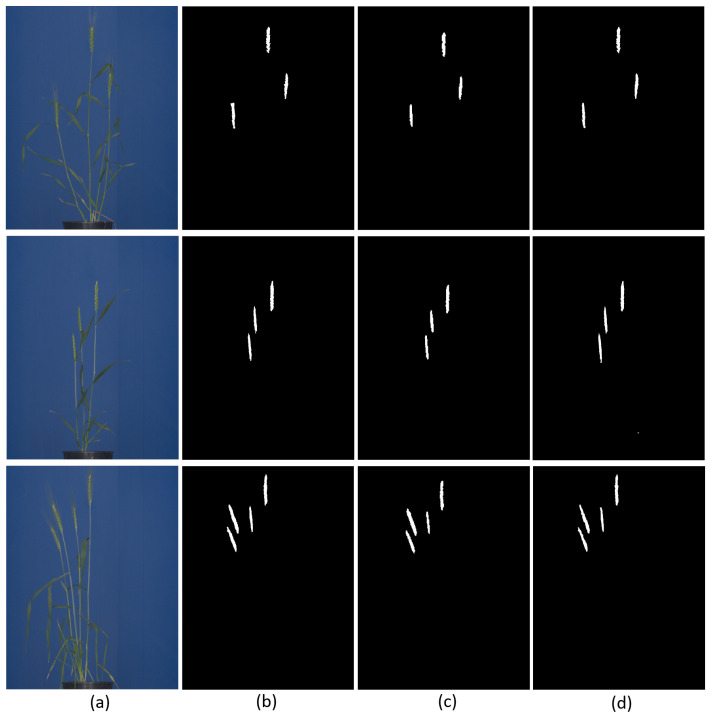
Examples of U-Net and DeepLabv3+ segmentation of spike images: (**a**) original test images, (**b**) ground truth binary segmentation of original images, and segmentation results predicted by (**c**) U-Net and (**d**) DeepLabv3+, respectively. The predominant inaccuracies in both NN models are associated with boundary pixels of spike followed false positive.

In total, 255 RGB images in the original image resolution of 2560 × 2976 were used for training and 43 for model evaluation. In this study, DeepLabv3+ was trained for 2000 epochs with a batch size of 6. The polynomial learning rate was used with weight decay of $1\times 10^{-4}$. The output stride for spatial convolution was kept at 16. The learning rate of the model was $2\times 10^{-3}$ to $1\times 10^{-5}$ with weight decay of $2\times 10^{-4}$ and momentum of 0.90. The evaluation metrics for in-training performance was mean IoU for the binary class labels, whereas net loss across the classes was computed from cross-entropy and weight decay loss. ResNet101 was used as the backbone for feature extraction. On the test set, DeepLabv3+ showed the highest aDC of 0.935 and Jaccard index of 0.922 among the three segmentation models. In segmentation, the DeepLabv3+ consumed more time/memory (11 GB) to train on GPU, followed by U-Net (8 GB) and then ANN (4 GB). Examples of spike segmentation using two best performing segmentation models, i.e., U-Net and DeepLabv3+, are shown in Figure 7.

### 3.3. Domain Adaptation Study

**Figure 8:**
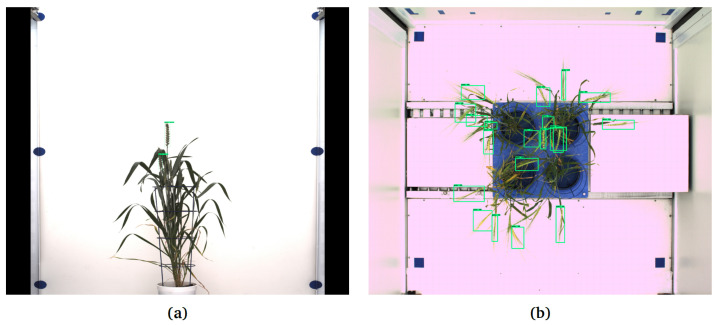
Examples of application of Faster-RCNN trained on data set in Table 2 for the detection of spikes of Central European wheat cultivars in images with different (white) background: (**a**) DNN failed to detect some spikes in the side view image, (**b**) early emergent spikes and some matured spike in the top view remained undetected.

To evaluate the generalizability of our spike detection/segmentation models, two independent image sets were analyzed:

- Barley and rye side view images that were acquired with the optical setup, including blue background photo chamber, viewpoint and lighting conditions as used for wheat cultivars. This image set is given by 37 images (10 barley and 27 rye) RGB visible light images containing 111 spikes in total. The longitudinal lengths of spikes in barley and rye were greater than those of wheat by a few centimeters (based on visual inspection).
- Two bushy Central European wheat cultivars (42 images, 21 from each cultivar) imaged using LemnaTec-Scanalyzer3D (LemnaTec GmbH, Aachen, Germany) at the IPK Gatersleben in side view, having on average 3 spikes per plant Figure 8a, and top view Figure 8b comprising 15 spikes in 21 images. A particular challenge of this data set is that the color fingerprint of spikes is very much similar to the remaining plant structures.

#### 3.3.1. Evaluation Tests with New Barley/Rye Images

**Figure 9:**
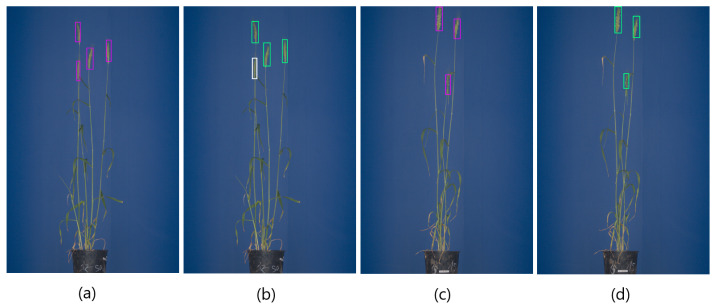
(**a**–**d**) shows detection examples of barley and rye spikes. White bounding boxes indicate spikes that were not detected by the DNN classifier in this particular image.

Evaluation tests with these new images showed that YOLOv4 outperforms Faster-RCNN and YOLOv3 measured with regard to the F1 score and $AP_{0.5}$ on the test set of barley and rye images. On the barley images, YOLOv4 achieved an F1 score of 0.92 and $AP_{0.5}$ of 0.88 followed by YOLOv3 with an F1 score of 0.91 and $AP_{0.5}$ of 0.85. Furthermore, we evaluated the rye images separately on F1 and $AP_{0.5}$. On the rye test images, YOLOv4 also performed the highest with an F1 score of 0.99 and $AP_{0.5}$ of 0.904, followed by YOLOv3 ($AP_{0.5}=0.870$) and Faster-RCNN ($AP_{0.5}=0.605$). The less accurate prediction of Faster-RCNN on the barley and rye is associated with false multiple spike detection (FP). The detection results of YOLOv4 and Faster-RCNN of barley and rye spikes are depicted in Figure 9a–d. The overview of model performance on the barley/rye data set is shown in Table 7.

**Figure 10:**
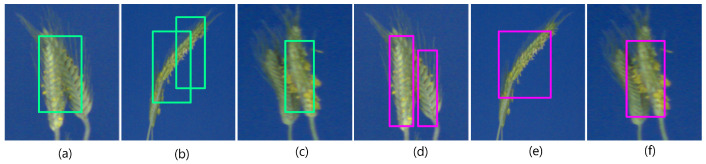
Overlapping/occluding spikes in barley/rye dataset: (**a**–**c**) Faster-RCNN failed to detect overlapping spikes as separate objects in the majority of cases and (**d**–**f**) YOLOv3, sometimes managed to separate occluding spikes as in (**d**).

In this case, the better performance of YOLOv4 is associated with non-maximal suppression of multiple bounding boxes on a single spike. When we further tested the detection DNNs on overlapping (partially occluded) spikes in the barley/rye test set, in most cases, Faster-RCNN produced multiple prediction or false positives, while YOLOv3 and its v4 variant performed well on it; see Figure 10. When U-Net and DeepLabv3+ were tested on barley and rye images, U-Net attained aDC of 0.31, whereas DeepLabv3+ showed an increase of 39% with aDC of 0.43.

#### 3.3.2. Evaluation Tests with Images from Another Phenotyping Facility

In addition to images of different grain plants from the same screening facility, the evaluation of spike detection models was performed with images from two bushy Central European wheat cultivars that were acquired from another plant phenotyping platform. These evaluation tests showed that the F1 score of Faster-RCNN on two cultivars was better (0.415) than YOLOv3/v4 (0.22) on bushy cultivars; see examples in Figure 8. While barley and rye images, such as those shown in Figure 9a–d, closely resembled wheat images that were used for the training of DNNs (Figure 4a–h), wheat images from the IPK Gatersleben exhibited quite different phenotypes, with multiple spikes emerging within a mass of leaves with the same color fingerprint as spikes; see Figure 8. The results of the DNN detection model performance on wheat images from another (IPK) screening facility are summarized in Table 7.

For these plants, Faster-RCNN turned out to perform better with $AP_{0.5}=0.41$ than YOLOv4 and YOLOv3, with $AP_{0.5}$ of 0.24 and 0.23, respectively; however, it could mainly detect spikes on the top of the plant (90%) and mostly failed on emerging spikes surrounded or occluded by leaves; see Figure 8a. Furthermore, DNNs detection models originally trained on side view images were exemplarily tested on top view images of central European wheat cultivars. Due to the enormous difference in illumination, the spatial orientation, optical appearance, projection area and overall shape of spikes in the top view differ from the side view images that were used for model training. Consequently, Faster-RCNN attained an $AP_{0.5}$ of 0.20 followed by YOLOv4 (0.14) and YOLOv3 (0.10) for this test set of top wheat images.

### 3.4. SpikeApp Demo Tool

**Figure 11:**
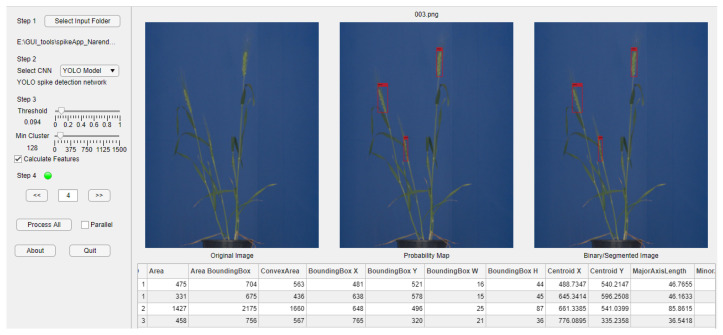
Screenshot of the SpikeApp tool for demonstration of DNN/ANN performance on detection, segmentation and phenotyping of grain spikes. On the left-hand side of the tool, the control and parameter section can be found, while on the right, the output area located. On the right, below the images, a table with the extracted features for all images is provided to the user for quick feedback.

Three out of six neural network models investigated in this study, namely, YOLOv3 for spike detection as well as ANN and U-Net for spike segmentation, were integrated into a GUI-based software tool (SpikeApp), not only demonstrating the performance of these three models, but also calculating over 70 phenotypic traits of detected spikes regions in terms of color, shape and textural descriptors. Figure 11 shows the screenshot of the SpikeApp, which can be downloaded along with example images from https://ag-ba.ipk-gatersleben.de/spikeapp.html (accessed on 1 November 2021, Gatersleben, Germany).

## 4. Discussion

This study aimed to quantitatively compare the performance of different neural network models trained on a particular set of images for the detection and segmentation of grain spikes in visible light greenhouse images acquired from the same as well as different phenotyping facilities. Therefore, the following observations were made. The predictive power of trained detection models certainly depends on optical properties of spike patterns and their position within the plant. Occluded/emergent as well as inner spikes appearing in the middle of a mass of leaves, present a more challenging problem for DNN models, compared to matured top spikes that were predominantly used in this and also previous works for model training. On images of reduced resolution, the accuracy of the DNNs decreased because of the loss in textural and geometric information. In particular, the best performing detection DNNs (YOLOv3/v4 and Faster-RCNN) achieved higher accuracy on matured top spikes, while for the group of inner and occluded/emergent spikes, the performance of Faster-RCNN was reduced. The application of DNN models trained on a particular set of side view wheat images to another crop cultivars (barley and rye acquired from the same phenotyping facility was associated with a relatively moderate reduction in the accuracy of spike detection. For improved performance of DNNs detection, the inclusion of different spike phenotypes in the training set is generally desirable. On the other hand, spikes from the YSYC test set were detected with 100% accuracy despite the fact that they have similar colors as the remaining plant biomass. In barley and rye, the most inaccuracies resulted from occluding/overlapping spikes. This problem likely cannot be solved by expanding the feature pool and requires separate handling. In contrast to images acquired from the same screening facility, the performance of detection models on phenotypically quite distant crop cultivars imaged in another facility was considerable worse. Therefore, side view spikes could be detected slightly better than spikes in the top view; however, this is not surprising in view of the larger differences between the optical appearance of spikes from side and top views. As a general conclusion from the above tests, the consideration of a significantly larger amount of manually annotated images, including different spike phenotypes, appears to be required in order to significantly improve the generalizability of DNN model predictions. Additionally, appropriate augmentation of existing ground truth data can be expected to improve the model performance. In this regard, it is remarkable that YOLOv4, which has the built-in image augmentation methods of Random Erase, CutMix and MixUp, showed the most robust performance by detection of occluded/emergent spikes. Summarizing the results of the spike detection tests, SSD shows the poorest performance, due to the lack of downscale feature extraction in small objects, as also observed in another study [31]. YOLOv4 deploys the feature extraction at three different scales, which improves spike detection compared to Faster-RCNN. In contrast to detection DNNs, segmentation models turned out to be more sensitive to phenotypic variations in plant and spike appearance. In previous works, conventional ANN approaches to spike segmentation were reported to achieve a relatively high accuracy of aDc > 0.95. However, in this study, the ANN framework from *Narisetti et al.* exhibited a rather moderate accuracy of aDC=0.76. We traced the reduced accuracy of the ANN framework back to differences between image sets used in previous and our studies. With aDC of 0.906 and 0.935, both U-Net and DeepLabv3+ models clearly outperformed the shallow ANN model by a direct comparison on the same image set, and exhibited relatively high segmentation accuracy by evaluation on both side view wheat images. However, when applied to other crop cultivars, the performance dropped to more than half, compared to the training data set. This indicates that significantly more variable ground truth data are required to achieve a more robust performance of spike segmentation models. Future improvements of segmentation DNNs can include the introduction of more classes for annotation of different background structures (photo chamber, plant canopy), which may improve accuracy of spike detection, particularly in cases where spikes exhibit similar color and texture fingerprints as the background. To mitigate the performance drop of segmentation DNNs on boundary regions of spike in unseen data, the neural network should have a broad feature map to accommodate the fine texture in the edge as well as the central part of a spike. In recent studies, to make DNNs more robust, one improvement was the introduction of weighting each channel (attention) in several layers to emphasize more on the informative channel and scale relevant feature of the object [34].

## 5. Conclusions

Our study showed that the performance of DNN models trained on a relatively modest set of ground truth images depends on the optical spike appearance (phenotype) as well as the spatial spike location within the plant in different crop cultivars. Detection DNNs showed an accurate and robust performance crossover of different crop cultivars, including wheat, barley and rye plants. From this observation, we conclude that DNNs trained on a particular set of plant images can, in general, be expected to show comparable performance by spike detection in other phenotypically similar crop cultivars. For the task of pixel-wise spike segmentation, DNN models, such as DeepLabv3+ and U-Net, showed superior performance compared to the conventional shallow ANN. Otherwise, segmentation DNNs showed accurate segmentation results only by application to images of the same cultivar phenotypes as in the training set. A particular challenge for DNN segmentation models seems to represent pixels on the spike boundary, as they exhibit particularly large variations in color and neighborhood properties, depending on the type of grain crops (e.g., grain with spikes emerging on the top of the plant vs. bushy plant phenotype; spike color, texture, size, shape); and scene illumination. From the results of this study, we conclude that a considerably larger set of different plant and spike phenotypes is required to achieve significantly more accurate segmentation of spikes. using DNN models. On the other hand, tested segmentation models (U-Net, DeepLabv3+) appear to be suitable for accurate spike segmentation in large image sets after training on a relatively modest amount of ground truth images. This constitutes them as being suitable tools, especially when processing large amounts of phenotypically similar images is the primary goal. In view of the limitations of different DNN methods, a combination of spike detection and segmentation approaches—in particular, YOLO and DeepLabv3+—appears to be a promising approach for improved analysis and phenotyping of spikes in images of different cereal crops. Finally, it should be stated that the above investigated neural network frameworks are principally not restricted to a particular task. Therefore, they can be expected to exhibit similar performance, advantages and shortcomings by application to the detection and segmentation of other plant structures. 

## Figures and Tables

**Table 1:** Summary of previous works on spike image analysis under field and greenhouse conditions, including the reference to publication, methods, accuracy measures/values as well as the application (field or greenhouse phenotyping). In different studies, different evaluation measures, including average precision (AP), accuracy of the confusion matrix (A) and/or F1-score (Dice coefficient), were used.

Publications	Methods	Measure	Value	Application
Tan et al. (2020)	Statistical analysis of morpho-colorimetric descriptors	AP	0.90	field
Alharbi et al. (2018)	Transforming raw plant images using color index of vegetation extraction (CIVE)	AP	0.90	field
Bi et al. (2010)	3-layer neural network to extract spike traits	AP	0.88	field
Misra et al. (2020)	Two cascaded feature networks	F1	0.97	field
Hasan et al. (2018)	R-CNN	AP/F1	0.93/0.95	field
Pound et al. (2017)	DNN	F1	0.89	field
Grillo et al. (2017)	Elliptic Fourier descriptors	AP	0.89	field
Qiongyan et al. (2017)	Artificial (shallow) neural network (ANN) approach to spike segmentation	A	0.92	greenhouse
Narisetti et al. (2020)	Extension of the ANN approach by Frangi line filter	A	0.98	greenhouse

**Table 2:** Overview of 19 Central European wheat cultivars used in this study for training and the evaluation of detection and segmentation methods. The remaining cultivars (nine) corresponding to genotype 11 include Pannonia NS, Tobak, Stephens, Cubus Bohemia, Midas, Genius, Fakir, Turandot, and Frisky.

Genotypes	Cultivar	Images
1	Avenue	45
2	Elly	14
3	IS Spirella	32
4	Amerigo	13
5	Manager	23
6	Pobeda	24
7	Jindra	23
8	Izvor	29
9	Timber	12
10	Ilona	22
11	Remaining cultivars	55
Total		292

**Table 3:** Summary of training and test image sets used for detection and segmentation tasks.

	Training Set					
**Total Images**	**GSGC**	**YSYC**	**Non-Spike**	**Test Set**	**View**	**Resolution**
292	203	27	4	58	side	2560 × 2976

**Table 4:** Summary of evaluation of spike detection DNN models on PASCAL VOC ($AP_{0.5}$) and COCO detection metrics ($AP_{0.5:0.95}$), respectively. Best results are highlighted in bold.

Detection DNNs	Backbone	Training Set/Aug.	${\mathrm{AP}}_{0.5}$	${\mathrm{AP}}_{0.75}$	${\mathrm{AP}}_{0.5:0.95}$
SSD	Inception resnet v2	234/none	0.780	0.551	0.470
YOLOv3	Darknet53	234/none	0.941	0.680	0.604
YOLOv4	CSPDarknet53	234/yes	0.941	0.700	0.610
Faster-RCNN	Inception v2	234/none	**0.950**	0.822	**0.660**

**Table 5:** Summary of detection model evaluation on matured top/inner vs. occluded/emergent spikes. Inner spikes include also occluded spikes. Probability, *Pr* is shown in the first column. $AP_{0.5}$ and $AP_{r}$:$AP_{0.5:0.95}$ are PASCAL VOC and COCO evaluation measures, respectively. The numbers of the top, occluded and inner spikes are 80, 27 and 45. The best results are highlighted in bold.

	Top Spikes: 80			Occluded/Emergent: 27			Inner Spikes: 45		
**Methods**	***Pr***	${\mathrm{AP}}_{0.5}$	${\mathrm{AP}}_{r}$	***Pr***	${\mathrm{AP}}_{0.5}$	${\mathrm{AP}}_{r}$	***Pr***	${\mathrm{AP}}_{0.5}$	${\mathrm{AP}}_{r}$
SSD	0.81	0.910	0.620	0.59	0.650	0.301	0.70	0.650	0.320
YOLOv3	0.97	0.999	0.708	0.74	0.750	0.450	0.96	0.890	0.500
YOLOv4	0.99	0.999	0.700	0.74	**0.805**	**0.550**	0.96	0.880	0.520
Faster-RCNN	0.99	0.999	**0.750**	0.79	0.800	0.532	0.96	**0.910**	**0.570**

**Table 6:** Summary of evaluation of spike segmentation models. The aDC score characterizes overlap between predicted plant/background labels and the binary ground truth labels as defined in Section 2.6. The U-Net and DeepLabv3+ training sets include 150 and 43 augmented images on a baseline data set of 234 images in total. Therefore, no augmentation was used by the training of ANN. The best results are shown in bold.

Segmentation Model	Backbone	Training Set/Aug.	aDC/m.F1	Jaccard Index
ANN	–	234/none	0.760	0.610
U-Net	VGG-16	384/150	0.906	0.840
DeepLabv3+	ResNet101	298/43	**0.935**	**0.922**

**Table 7:** Summary of detection/segmentation DNNs performance evaluation on barley/rye spikes and bushy wheat cultivars. The best results, evaluated on *F*1 and *AP* are compared row-wise for barley/rye and bushy wheat cultivars and shown in bold. On barley/rye dataset, YOLOv4 performed better than Faster-RCNN, whereas DeepLabv3+ showed higher aDC, having more accurate spike boundaries. On Bushy wheat cultivars, DNNs failed to segment spikes. On side view bushy wheat cultivar images, Faster-RCNN achieved higher *AP* compared to top view wheat cultivar.

			Barley/Rye Dataset				Bushy Wheat Cultivars			
**Methods**	**Backbone**	**aDC**	***F*1**_**barley**_	***AP***_**0.5:barley**_	***F*1**_**rye**_	***AP***_**0.5:rye**_	***F*1**_**top**_	***AP***_**top**_	***F*1**_**side**_	***AP***_**side**_
YOLOv3	Darknet53	-	0.91	0.850	0.99	0.870	0.15	0.100	0.25	0.233
YOLOv4	CSPDarknet53	-	**0.92**	**0.880**	**0.99**	**0.904**	0.20	0.140	0.30	0.240
Faster-RCNN	Inception v2	-	0.80	0.690	0.79	0.650	**0.28**	**0.205**	**0.55**	**0.410**
U-Net	VGG-16	0.310	-	-	-	-	-	-	-	-
DeepLabv3+	ResNet101	**0.430**	-	-	-	-	-	-	-	-

## Data Availability

Links to data and tools described in this work are provided with the manuscript.
